# The Impact of Psychopharmacology on Contemporary Clinical Psychiatry

**DOI:** 10.1177/070674371405900803

**Published:** 2014-08

**Authors:** Gustavo H Vázquez

**Affiliations:** 1 Professor and Director, Department of Neuroscience, University of Palermo, Buenos Aires, Argentina.

**Keywords:** clinical psychiatry, psychiatric diagnosis, psychiatric treatments

## Abstract

Clinical psychiatric evaluations of patients have changed dramatically in recent decades. Both initial assessments and follow-up visits have become brief and superficial, focused on searching for categorical diagnostic criteria from checklists, with limited inquiry into patient-reported symptomatic status and tolerability of treatments. The virtually exclusive therapeutic task has become selecting a plausible psychotropic, usually based on expert consensus guidelines. These guidelines and practice patterns rest mainly on published monotherapy trials that may or may not be applicable to particular patients but are having a profound impact, not only on modern psychiatric practice but also on psychiatric education, research, and theory.

## Abstract (fr)

Les évaluations psychiatriques cliniques des patients ont changé radicalement ces dernières décennies. Tant les évaluations initiales que les visites de suivi sont devenues brèves et superficielles, axées sur la recherche de critères diagnostiques catégorisés dans des listes de vérification, sans beaucoup de recherche sur le statut symptomatique déclaré par le patient et sa tolérabilité aux traitements. La tâche thérapeutique presque essentielle est devenue la sélection d’un psychotrope plausible, fondée sur des lignes directrices issues d’un consensus d’experts. Ces lignes directrices et ces modèles de pratique reposent principalement sur des essais publiés de monothérapie qui peuvent ou non être applicables à des patients particuliers, mais qui ont un effet profond non seulement sur la pratique psychiatrique moderne, mais aussi sur l’éducation, la recherche et la théorie psychiatriques.

In visits to North America, as well as in experiences in South America, I have been struck by observations of changes in clinical psychiatric interviews in recent years, in various settings. Typically, they are very brief, involve minimal personal interaction, and are marked by the pervasive intrusion of ever-present computers. The content of interviews, particularly during follow-up visits, often consists of routine and superficial questions from a memorized checklist, completed within a few minutes, followed by encouragement to continue the same medicines and instruction to make an appointment to return for a similar visit in perhaps 2 months. Even initial assessments of new patients are remarkably brief and focused on seeking criteria (again from a memorized checklist) to generate a categorical diagnosis based on DSM or the ICD criteria that are considered standard.^1^ Once a patient has been placed in one or more tentative diagnostic pigeonholes, there inevitably follows a prescription, as the treatment selected is nearly always medicinal. As noted by Dr David M Gardner^2^ in his In Review paper in this issue, at best, treatment selection appears to be based on guidelines derived by the consensus of so-called experts, rather than the personal expertise of the prescribing clinician. These phenomena are not unique to the Americas, and appear to be commonplace internationally.

In the contemporary rush toward genetic, molecular, and imaging studies in the elusive search for diagnostic and therapeutic answers to pressing but unanswered clinical questions, psychopathology appears increasingly to be considered a nonscientific method, even in European academic centres that formerly led such inquiry.^3^ More generally, there seems to be a growing lack of interest in clinical details of the experiences of individual psychiatric patients, or in the impact of both symptoms and life circumstances on patients and their families. This trend has profound implications for adequate diagnostic, prognostic, and therapeutic understanding of patients as individuals with an illness and for giving them competent care.^4^–^7^

To recapitulate, current trends in clinical psychiatry include the following:

1. increasingly brief clinical assessments;
2. reliance on simplified and potentially misleading diagnostic schemes based largely on symptom checklists and somewhat arbitrarily rigid criteria for growing numbers of proposed but inadequately established psychiatric disorders;
3. the increasingly routine assumption that picking the right psychotropic is the main therapeutic task; and then,
4. brief and infrequent follow-up encounters.

It is hard to avoid the impression that these trends are encouraged by the domination of psychiatric therapeutics by use of medicinal treatments. Their benefits, appropriately, are highly valued but typically limited, and their adverse effects are often less than trivial; rarely do they represent adequate, let alone comprehensive, clinical care. In turn, the clinical approaches noted surely are strongly encouraged by efforts to limit the costs of clinical care, often in the service of greater efficiency. These trends are having a profound impact, not only on the nature of modern psychiatric practice but also on psychiatric education, research, and theory, as noted by Dr Ross J Baldessarini^8^ in his Guest Editorial. Adequate and fair assessment of such trends is complicated. Valuable, even revolutionarily, improvements in the treatment of patients with major psychiatric disorders have been achieved by generally effective, reasonably well-tolerated, and usually affordable psychotropics. However, the question remains, are these gains being compromised by largely unanticipated tendencies toward more impersonal and less comprehensive care of individual patients with complex clinical problems?

Psychiatry may be particularly vulnerable to pressures that encourage briefer clinical encounters, reliance on diagnostic checklists, and treatment largely limited to prescription-writing. Such trends affect general medicine as well, but seem to be less effectively resisted, and more disruptive to traditional clinical practices in psychiatry. Other medical and surgical specialties are considered to deal more with acute life-and-death issues, and to be worthy of greater proportions of available resources. In reality, however, there is an abundance of disabling, life-threatening and -shortening aspects of major psychiatric illnesses, including high rates of suicide, especially in the young, as well as greater mortality with general medical disorders in older psychiatric patients.^9^–^11^ Sometimes increased mortality has even been associated with the use of prescribed psychotropics.^12^–^14^

Since the 1950s, psychiatry has been waiting for the striking and impressive advances in neuroscience to transform psychiatry into a more medical or biological discipline. Such efforts seek to regain greater affiliation to general medicine, and perhaps ultimately to replace mental illness with brain disease. Although there have been major advances in the past half-century in clinical and basic psychopharmacology, and stunning advances in basic and clinical neuroscience generally, a neurobiological foundation of major mental illnesses, specifically as a means of improving diagnosis and prognosis, and for guiding development of innovative treatments, is still awaited.^15^–^19^

Another notable recent trend is that innovation in psychopharmacologically based therapeutics has slowed substantially. Laboratory-based, basic psychopharmacology and neuroscience continue apace, but fundamental innovation leading to new drug products for the treatment of mental illnesses that are superior in effectiveness and tolerability, or fundamentally different from older drugs, has remained elusive. This circumstance has led increasing numbers of pharmaceutical corporations to shift their efforts and investments to other areas or to disappear through mergers. Companies that continue in psychopharmacology often rely on modest variations on old pharmacodynamic actions and known drugs, including marketing of isomers or active metabolites, or agents designed and selected to mimic previously successful products rather than arising from fundamental and scientifically predicted and guided innovation.^14^ In turn, the lack of compelling and relevant pathophysiologies, let alone etiologies, of most psychiatric disorders limits efforts at rational therapeutic innovation and encourages reliance on principles arising from largely serendipitous earlier discoveries.

**Highlights**

- There is a growing lack of interest in phenomenological aspects and clinical details of the experiences of individual psychiatric patients.
- Contemporary psychiatric therapeutics is based mainly on pharmacotherapy dictated by guidelines and algorithms arising from corporate-sponsored drug trials designed for commercial purposes.

Another apparent corollary of the difficulties of therapeutic innovation in psychiatry is reflected in the current state of psychiatric nosology. There is pressure to maximize potential markets by retaining overly broad diagnostic concepts, such as major depressive disorder, as well as by the implausible proliferation of psychiatric disorders to several hundred in recent editions of the DSM. Moreover, some psychotropics are promoted for a growing range of conditions. Examples include expanding indications of antidepressants to various anxiety-related disorders, and even some somatic conditions,^20^,^21^ and of antipsychotics to the treatment of mania and depression.^22^

In addition to broadening of drug indications in pursuit of industrial marketing efforts as well as hoped-for clinical benefits, there also are risks of overgeneralizing about classes of drugs. For example, dividing antipsychotics into typical and atypical agents, based on their relative risks of some adverse neurological effects, is unsatisfactory and can be misleading: neither drug group is homogeneous, based on chemistry, pharmacodynamics, or on beneficial or adverse clinical effects.^14^,^23^,^24^ Moreover, broadening of potential indications for particular types of psychotropics can contribute to degrading the relation of drug selection to diagnosis and to adequate understanding of typically complex, individual psychiatric patients. I have heard trainees comment—only partly in jest—that detailed and individualized clinical assessments represent wasted time and effort, in that the choice of treatment for virtually any psychotic disorder, including schizophrenia, acute psychosis, mania, many types of depression, and perhaps even some anxiety disorders, is the use of a modern antipsychotic.

Such conclusions and practices often appear to be supported by available evidence, as interpreted by regulatory agencies, aggressively promoted in industrial marketing campaigns, accepted by respected journals, and recommended in expert guidelines.^22^,^25^ Increasingly, however, evidence of the clinical value and safety of a drug arises from studies aimed less at identifying ideal clinical applications and limitations than at supporting the licensing and marketing aims of pharmaceutical manufacturers. Such aims are entirely legitimate and to be expected, but have only limited bearing on clinical decision making and therapeutic practice. Subjects studied in experimental treatment trials often are highly selected and sometimes poorly representative of many clinically encountered patients. Moreover, even well-designed and -conducted, and fairly analyzed and reported, RCTs yield averaged findings that may or may not apply reliably to subgroups or to more complex individual patients.^26^

Moreover, much of the evidence of clinical effectiveness in a particular target population is based on short-term studies, sometimes with relatively brief continuation. All too often, effective treatments are discontinued after partial clinical recovery, resulting in a relapse that is commonly misinterpreted as proof of long-term prophylactic benefit.^14^ In addition—again based on statements of expert authorities— long-term care is often considered adequate, with continued use of an initially prescribed drug or perhaps with serial trials of other agents of similar type. Such oversimplification of clinical practice and avoiding detailed, individualized, and flexible longitudinal assessments of patients with typically complex, changeable, and only partially treatment-responsive illnesses can only further degrade the quality of psychiatric care.

Despite wide distribution of reports arising from RCTs sponsored, designed, and analyzed by manufacturers of products studied, a striking lack of critical and clinically relevant information arises from them with which to evaluate or optimize clinical applications of psychopharmacological treatments for individual patients. Even academic reviews and assessments of available treatments are heavily constrained by relying on clinical therapeutics research findings that are almost entirely supported by manufacturers of products tested and only partly relevant to clinical practice. Potential RCTs participants typically are excluded if they have multiple medical and psychiatric illnesses, substance abuse, poverty or homelessness, engage in risky behaviours, or have other characteristics commonly encountered in the real world of everyday practice. In addition, the assessments employed in treatment RCTs almost always involve changes in scores on standardized symptom-rating scales rather than evaluations of improved functioning, overall health, and patient satisfaction. Adverse effects of treatments continue to be identified almost entirely by passively acquired reports from study participants or incidental observations by their clinicians, rather than by prospective, preplanned, systematic, and explicit assessments, with a high risk of undercounting uncommon adverse events. Again, outcome measurements in RCTs characteristically lack a high degree of relevance to the complex clinical problems presented by most psychiatric patients or their responses to treatment, and tend to submerge analyses of potentially important subgroups into broad, generalized, averaged conclusions that are far more applicable to marketing than clinical aims.

Moreover, findings from even well-designed and -analyzed RCTs represent average trends that are typically combined by methods of meta-analysis (averages of averages) in which each trial counts as a single observation. Almost always, such data analyses fail to distinguish effectively or convincingly one marketed product from another of similar type by efficacy or safety,^14^ and say nothing about clinically nonaverage patients, or those from dissimilar sociocultural backgrounds.^27^ Data included in summary analyses most often involve findings favourable to particular commercial products.^28^,^29^ Efforts to include negative or unfavourable, often unpublished, study findings are sometimes made, but usually inconsistently and incompletely, or without critical peer review.^30^

An additional shortcoming of scientific assessments of treatments is that clinical practice typically, and increasingly, involves empirically applied combinations of treatments aimed at dealing with the substantial proportions of patients whose responses to monotherapies are unsatisfactory or short-lived. This practice of empirical polytherapy is also encouraged by the concept of comorbidity, or presence of separately diagnosed clinical disorders, which may, instead, be manifestations of a single illness.^31^ Rarely are specific combination treatments evaluated for their relative effectiveness, safety, and cost, compared with monotherapies, or are monotherapies themselves tested for effects on various conditions that are considered comorbid.^31^–^34^

Traditionally, both individual and meta-analyzed RCTs have avoided consideration of subgroups that may respond particularly well or poorly, or have especially low or high risks of adverse responses. This circumstance may sometimes be motivated by fear of market segmentation, and commercial longing for large, but oversimplified, markets. Paradoxically, emphasis on broad indications suggests a lack of appreciation of potential marketing advantages to be gained by being distinguished by proof (with regulatory recognition) of being particularly effective for specific subgroups of patients. Such distinctions can matter for marketing as well as for clinical care as most drugs within a class appear to be quite similar in average efficacy and tolerability—at least as evaluated with pooled, averaged responses.

In conclusion, several trends in contemporary clinical practice involving psychotropics for patients with psychiatric disorders are noted. They include increasingly brief and frankly superficial clinical assessments, disincentives to invest in deeper understanding of individual patients, reliance on simplistic checklists for categorical diagnosis, and as a substitute for individualized clinical evaluation, along with treatment based on pharmacotherapy dictated by guidelines and algorithms that are not likely to be developed independent of manufacturers of products considered. These trends, in large part, are encouraged by apparent therapeutic efficiency of psychotropics in an atmosphere of cost-containment. They have led to an overall decline of clinical curiosity—a regrettable and evidently ubiquitous characteristic of contemporary clinical practice in psychiatry internationally. Modern pharmacotherapy has had a profound, but mixed, impact on clinical practice and on psychiatric education and training. It includes unprecedented therapeutic gains while encouraging brief contacts and relatively superficial clinical understanding of individual patients. These trends have profoundly discouraged formerly standard efforts to exercise real interest aimed at understanding complex human problems, and to work imaginatively, flexibly, and adaptively to develop, modify, and pursue adequate clinical care for individual patients whose needs are likely to change over time.

## Abbreviations

DSM: Diagnostic and Statistical Manual of Mental Disorders
ICD: International Classification of Diseases
RCT: randomized controlled trials
